# Some Novel Insights on HPV16 Related Cervical Cancer Pathogenesis Based on Analyses of LCR Methylation, Viral Load, E7 and E2/E4 Expressions

**DOI:** 10.1371/journal.pone.0044678

**Published:** 2012-09-06

**Authors:** Damayanti Das Ghosh, Bornali Bhattacharjee, Shrinka Sen, Laikangbam Premi, Indranil Mukhopadhyay, Rahul Roy Chowdhury, Sudipta Roy, Sharmila Sengupta

**Affiliations:** 1 Human Genetics Unit, Indian Statistical Institute, Kolkata, India; 2 Department of Gynecology, Saroj Gupta Cancer Centre and Research Institute, Kolkata, India; 3 Department of Pathology, Suraksha Diagnostics Private Limited, Kolkata, India; 4 National Institute of Biomedical Genomics, Kalyani, Dist. Nadia, West Bengal, India; University Magna Graecia, Italy

## Abstract

This study was undertaken to decipher the interdependent roles of (i) methylation within E2 binding site I and II (E2BS-I/II) and replication origin (nt 7862) in the long control region (LCR), (ii) expression of viral oncogene *E7*, (iii) expression of the transcript (*E7-E1∧E4)* that encodes E2 repressor protein and (iv) viral load, in human papillomavirus 16 (HPV16) related cervical cancer (CaCx) pathogenesis. The results revealed over-representation (p<0.001) of methylation at nucleotide 58 of E2BS-I among *E2*-intact CaCx cases compared to *E2*-disrupted cases. Bisulphite sequencing of LCR revealed overrepresentation of methylation at nucleotide 58 or other CpGs in E2BS-I/II, among *E2*-intact cases than *E2*-disrupted cases and lack of methylation at replication origin in case of both. The viral transcript (*E7-E1∧E4*) that produces the repressor E2 was analyzed by APOT (amplification of papillomavirus oncogenic transcript)-coupled-quantitative-RT-PCR (of *E7* and *E4* genes) to distinguish episomal (pure or concomitant with integrated) from purely integrated viral genomes based on the ratio, *E7* C_T_/*E4* C_T_. Relative quantification based on comparative C_T_ (theshold cycle) method revealed 75.087 folds higher *E7* mRNA expression in episomal cases over purely integrated cases. Viral load and *E2* gene copy numbers were negatively correlated with *E7* C_T_ (p = 0.007) and *E2* C_T_ (p<0.0001), respectively, each normalized with *ACTB* C_T_, among episomal cases only. The *k*-means clustering analysis considering *E7* C_T_ from APOT-coupled-quantitative-RT-PCR assay, in conjunction with viral load, revealed immense heterogeneity among the HPV16 positive CaCx cases portraying integrated viral genomes. The findings provide novel insights into HPV16 related CaCx pathogenesis and highlight that CaCx cases that harbour episomal HPV16 genomes with intact *E2* are likely to be distinct biologically, from the purely integrated viral genomes in terms of host genes and/or pathways involved in cervical carcinogenesis.

## Introduction

HPV16 appears to be the most common high risk HPV type identified in CaCx cases, precancerous cervical lesions, and in cytologically normal cervical samples [1], [2]. In India among the HPV positive CaCx cases, majority is HPV16 DNA positive [3], [4].

It is established that although the HPV genome exists in the episomal form in low-grade lesions, the viral genome gets integrated, with increasing grades of lesion [5]. Despite this observation, analysis of the association of HPV infections with CaCx development reveals the presence of both integrated as well as episomal forms of HPV, particularly HPV16, in CaCx cases [6]. Our observations have shown that *E2* gene disruption is significantly overrepresented among CaCx cases compared to controls [7]. However, over 60% of the CaCx cases harbor intact *E2*, despite the fact that HPV16 E2 protein negatively regulates transcription of the *E6* and *E7* genes [7], [8]. This prompted us to explore new paradigms of cervical carcinogenesis, which would offer insights into mechanisms involved in sustained *E6*/*E7* mRNA expression even with the *E2* gene intact, i.e. presence of concomitant viral genomes (both episomal and integrated) or purely episomal viral genomes [7], [9]–[11].

Based on a comparison between fifteen HPV16 positive cytologically normal and fifty-seven HPV16 positive CaCx cases with intact *E2* gene, we proposed that loss of E2 repressor activity in such cases, predominantly of the E-lineage, could be attributable to CpG methylation at nucleotide 58 within E2 binding site I (E2BS-I) next to the p97 promoter, thus attenuating the binding of E2 to this site [7], [9]. We subsequently observed that viral load of *E2*-intact cases was significantly higher compared to those with disrupted *E2* gene. This further prompted us to interpret that viral load in association with *E2*-status, might be of causal relevance in CaCx pathogenesis [12]. The biological plausibility of such observations is likely to be supported by the fact that E2 protein enhances viral DNA replication by interacting with the viral replication factor E1 and recruiting it to the origin of replication [13]–[15] and also plays a more direct role in replication facilitating viral genome segregation by tethering the viral genomes to host mitotic chromosomes [16]–[17].

We undertook the present study, focusing on HPV16 positive E2 intact and E2 disrupted CaCx cases initially, followed by their classification into episomal and integrated forms, to determine the interdependent roles of (i) methylation within E2 binding sites I and II (E2BS-I/II) and replication origin in the LCR, (ii) expression of viral oncogene *E7*, (iii) expression of the transcript (*E7-E1∧E4)* that encodes E2 repressor protein and (iv) viral load, in HPV16 related CaCx pathogenesis. Our study provided novel insights into alternative mechanisms of loss of E2 repressor activity, which could be related to E2BS-I/II methylation, presence of the transcript (*E7-E1∧E4*) that encodes E2 repressor protein, high viral load and *E7* expression among HPV16 positive CaCx cases with episomal (pure or concomitant with integrated) viral genomes and not among the cases with purely integrated viral genomes. In this study, we further identified by employing a novel technique, APOT-coupled-quantitative-RT-PCR, that there was immense diversity among the HPV16 positive CaCx cases both in terms of viral copy numbers and *E7* expression levels.

## Materials and Methods

### Samples and subjects

The samples used for this study were nested to an ongoing natural cohort study [7], [11], [12]. The HPV16 positive malignant samples (N = 184; histopathologically confirmed invasive squamous cell carcinomas and clinically diagnosed as tumour stage III and above as per FIGO classification) were derived from married subjects, attending a cancer referral hospital (Saroj Gupta Cancer Centre and Research Institute, South 24 Parganas, West Bengal, India). The samples (fresh biopsy tissues) were collected from the participants with written informed consent approved by the institutional ethical committee for human experimentation of the Indian Statistical Institute. Details regarding subjects, samples, DNA isolation, PCR-based HPV16 detection, determination of *E2* disruption status by overlapping DNA-based PCR and estimation of viral load are described elsewhere [12]. Out of the 184 cases, number of samples harboring intact *E2* was 140, and that having disrupted *E2* was 44.

### Determination of methylation status of HPV16 DNA by restriction enzyme digestion and PCR

In *E2*-intact cases, methylation status of the E2BS-I [50-ACCGAAA***CCGG***T-61] and also the region from LCR to *E6* (LCR-*E6*) were determined by restriction-digestion with *HpaII/MspI* and *McrBC* enzymes (New England Biolabs), respectively, and analyzed by PCR, following published protocols [9]. Approximately 20% of the methylated (based on *McrBC* restriction digestion) cases were randomly selected from each of *E2*-intact (30/140) and *E2*-disrupted (9/44) categories for bisulphite sequencing, using the primer pair 5′-TAA GGT TTA AAT TTT TAA GGT TAA TTA AAT-3′ and 5′-ATC CTA AAA CAT TAC AAT TCT CTT TTA ATA-3′ covering positions 7748-115 [18].

### RNA isolation and reverse transcription

Total RNAs, from 41 cancer samples, were isolated, purified and treated with DNase I using the Qiagen RNeasy kit following the manufacturer's protocol. One microgram of total RNA was reverse-transcribed using 200U of M-MuLV reverse transcriptase (Fermentas) in a 20 µl reaction containing 5X RT buffer [Fermentas; 250 mM Tris-HCl (pH 8.3 at 25°C), 250 mM KCl, 20 mM MgCl_2_, 50 mM DTT], 10 mM dNTP, 0.1 M DTT, 20U RNase Inhibitor and 400 ng of (dT)17-P3 primer. The mixture containing RNA and primers was heated at 70°C (10 minutes) and chilled (10 minutes) before addition of 5X buffer, 0.1 M DTT and RNase inhibitor, and then, incubated at 25°C (2 minutes), followed by addition of reverse transcriptase and subsequent incubations at 25°C (10 minutes) and 42°C (60 minutes). The reaction was inactivated at 70°C (15 minutes).

### Confirmation of physical status of HPV16 genomes and presence of the viral transcript, E7-E1∧E4, in CaCx cases, by APOT-coupled-quantitative-RT-PCR assay – a novel technique

#### Concept of APOT-coupled-quantitative-RT-PCR assay

The APOT assay was used primarily to get amplified products of the entire region from *E7* to *E2* using total cDNA formed with an oligo-dT primer (dT)17-P3, where P3 was an adaptor sequence (following the principle of 3′ RACE) [19]. Instead of the subsequent way of confirming intactness of viral genomes by Southern hybridization, nested PCRs with separate RT-PCR (TaqMan) primer-probe sets for *E7* and *E4* were designed in this study to check the intactness of viral genomes based on quantitative difference between *E7* and *E4* transcription levels.

*E7* is always (both in episomal and integrated conditions) found to be intact owing to its role in oncogenicity, and it does not harbour any splice-junction site. So, quantification of the *E7* transcription from the viral cDNA pool could mark viral presence irrespective of its genomic status (episomal or integrated). On the other hand, *E2* coding the intact repressor retains *E4,* which in turn holds the hinge-coding region of E2 protein. Hinge-coding region reportedly harbors majority of the disruption sites for viral integration into the host genome [20]. Thus, quantification of the hinge-coding region within *E4* from the viral cDNA pool, could mark presence of intact *E2*. So probe-primer sets were designed for *E7* and *E4* for TaqMan-based qRT-PCR on the APOT-PCR products. Differential amplification of *E7* and *E4* in episomes and integrates could be indicated by differences in C_T_-values of *E7*- and *E4*-specific qRT-PCR.

#### APOT-PCR

The first PCR with P1 (5′-CGG ACA GAG CCC ATT ACA AT-3′) and P3 (5′-GAC TCG AGT CGA CAT CG-3′) and the nested second PCR with P2 (5′-CCT TTT GTT GCA AGT GTG ACT CTA CG-3′) and (dT)17-P3 (5′-GAC TCG AGT CGA CAT CGA TTTTTTTTTTTTTTTT-3′) were performed on 41 CaCx cases following published protocols [19]. The PCR products were checked by agarose gel (1.5%) electrophoresis of P2 - (dT)17-P3 products.

#### TaqMan-based quantitative RT-PCR of E7 and E4 on APOT-PCR products

The reaction mix (10 µl) of *E7* and *E4* duplex qRT-PCR contained 2X TaqMan® Universal PCR Master Mix (Applied Biosystems), 3 ng primers and 2 µM probe. The primer-probe for HPV16 *E7* (forward: 5′- AAG TGT GAC TCT ACG CTT CGG TT -3′; reverse: -5′ GCC CAT TAA CAG GTC TTC CAA A- 3′; probe: 5′- FAM-TGC GTA CAA AGC ACA CAC GTA GAC ATT CGT A-BHQ -3′) and HPV16 *E4* (forward: 5′- CTT GGG CAC CGA AGA AAC AC -3′; reverse: 5′-GAT TGG AGC ACT GTC CAC TGA GT -3′; probe: 5′-Vic-ACG ACT ATC CAG CGA CC-BHQ -3′) produced 78 bp and 118 bp amplicons, respectively. The real time PCR program included UNG-activation at 50°C for 2 minutes, initial denaturation at 95°C for 10 minutes, followed by 40 cycles of denaturation at 95°C for 15 seconds and annealing at 60°C for 1 minute. The PCR-controls were NTC (non-template control) as well as separate aliquots from reverse transcription reactions with (i) all reagents except mRNA, (ii) mRNA and all reagents but no reverse transcriptase, and (iii) HPV-negative cellular mRNA. The duplex assay was performed at least twice, with three replicates per sample in each assay.

### PCR of GAPDH and TP53 to confirm presence of mRNA and absence of DNA

Presence of mRNA was confirmed by PCR with primers spanning exon-exon junction of *GAPDH*. The reaction volume of 10 µl contained 1.25 mM MgCl_2_, 100 µM dNTP, 1 Unit of AmpliTaq Gold® DNA polymerase (Applied Biosystems) and 50 ng primers (forward: 5′-CAG CCT CAA GAT CAT CAG CA-3′; reverse: 5′-TGT GGT CAT GAG TCC TTC CA-3′) and 1 µl cDNA in 10X AmpliTaq Gold® buffer (Applied Biosystems). The PCR program included 40 cycles of denaturation at 94°C, annealing at 55°C and elongation at 72°C, each for 1 minute, along with initial denaturation for 8 minutes at 95°C and final elongation for 5 minutes at 72°C. Presence of 106 bp amplicon was checked in 2.5% agarose gel.

Absence of DNA-contamination was confirmed by PCR with primers spanning introns of *TP53*. The reaction volume of 20 µl contained 2 mM MgCl_2_, 50 µM dNTP, 1 Unit of AmpliTaq Gold® DNA polymerase (Applied Biosystems), 40 ng primers (forward: 5′-CCT GAA AAC AAC GTT CTG GTA A-3′; reverse: 5′-GCA TTG AAG TCT CAT GGA AG-3′) and 2 µl cDNA in 10X AmpliTaq Gold® buffer (Applied Biosystems). The PCR program included 35 cycles of denaturation at 94°C, annealing at 56°C and elongation at 72°C, each for 1 minute, along with initial denaturation for 8 minutes at 95°C and final elongation for 5 minutes at 72°C. Presence of 448 bp amplicon was checked in 1.5% agarose gel.

### Relative quantification of *E7* and *E2* mRNA expression by qRT- PCR (TaqMan)

*E7* and *E2* gene expressions were quantified among a subset of 30 samples, of which 17 were episomal (pure or concomitant) and 13 were purely integrated by qRT-PCR (relative quantification with comparative C_T_ method). E7 expression was quantified by using the same primer-probe set as in APOT-coupled-quantitative-RT-PCR assay [*21*]. *E2 *expression was estimated from 82 bp amplicon by E2-specific primer-probe [forward: 5′ AAC GAA GTA TCC TCT CCT GAA ATT ATT AG 3′; reverse: 5′ CCA AGG CGA CGG CTT TG 3′; probe: 5′ (BODIPYR6G)-CAC CCC GCC GCG ACC CAT A-(DQ) 3′]. The reaction mixture (10 µl) contained 2 µl cDNA, 50 ng primers, 0.1 µM probes and 2X TaqMan® Universal PCR Master Mix, (Applied Biosystems). Human ACTB Endogenous Control (VIC/MGB Probe, Primer Limited) (Applied Biosystems) (amplicon size = 171 bp) was used as normalizer. The PCR-program and the controls used were same as in APOT-coupled-quantitative-RT-PCR assay. The assays, with three replicates per sample, were repeated twice.

### Statistical Analyses

Kolmogorov-Smirnov test was performed to identify whether the test-variables like viral copy numbers, expressions of housekeeping gene and viral genes followed normal distribution. Independent 2-sample t-test and Mann-Whitney U test were used to identify association with disease phenotype, respectively, for variables that followed normal distribution and those that did not. Chi-squared analysis was performed to test for association of methylation within E2BS-I with *E2* disruption-status among the CaCx samples. The *k*-means clustering algorithm (*k* = number of clusters) was used to categorize different forms of purely integrated viral genomes. All analyses were performed using software package, SPSS for windows v16.0.

## Results

### Methylation status of the LCR and the E2BS-I (nucleotide 58) of HPV16 positive CaCx cases harboring intact or disrupted *E2*

Subsequent to our earlier study [9] we further investigated the role of CpG methylation at nucleotide (nt) 58 within E2 binding site I (E2BS-I), in CaCx pathogenesis by a direct comparison between HPV16 positive CaCx cases harboring viral genomes with intact or disrupted E2 gene using an expanded set of one hundred and eighty-four CaCx samples. Restriction digestion (*HpaII/MspI*) and subsequent PCR analysis revealed methylation at E2BS-I (nucleotide 58), proximal to p97 promoter within the LCR, to be significantly higher (p<0.001) among *E2*-intact cases (69/140; 49.28%) compared to *E2*-disrupted cases (5/44; 11.36%).

**Figure 1:**
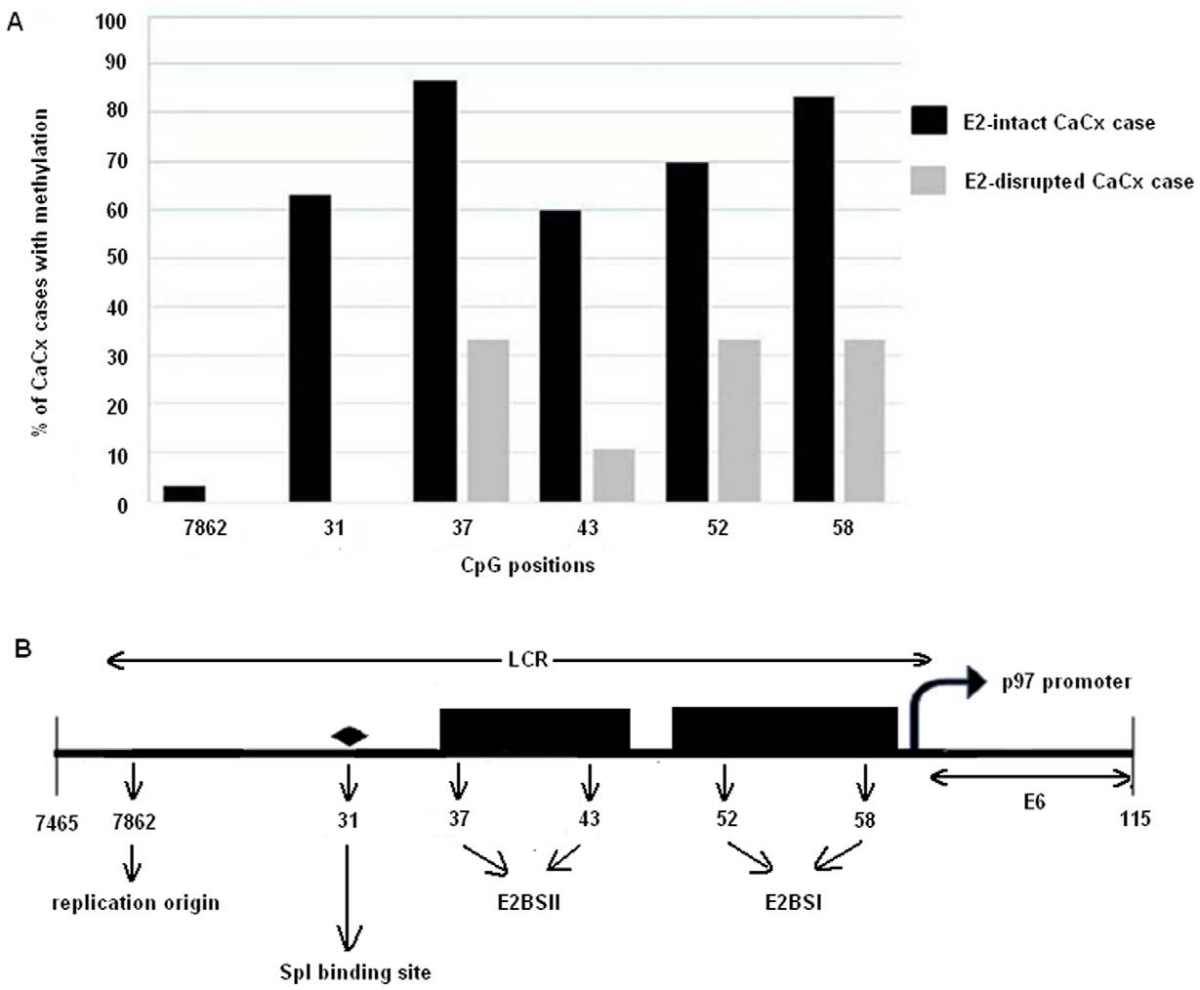
Methylation analysis. (**A**) Bar diagram comparing percentage of samples methylated at different CpGs within the LCR of HPV16 genome between *E2*-intact and *E2*-disrupted samples. (**B**) Diagrammatic representation of the mentioned CpG positions within the LCR: 7862 – Viral replication origin; 31 – SpI binding site; 37 and 43 – E2BS II; 52 and 58 – E2BS I.

Bisulphite-sequencing analysis of LCR DNA from 39 CaCx cases confirmed that among *E2*-intact cases (n = 30), methylation was more prominent (Figure 1) at nucleotide positions 31 (SpI binding site, 63.33%), 37 and 43 (E2BS-II, 86.66% and 60%, respectively), 52 and 58 (E2BS-I, 70% and 83.33%, respectively) compared to the viral origin of replication (position 7862, 3.3%). Moreover, compared to *E2*-intact cases, *E2*-disrupted cases (n = 9) portrayed no methylation at the positions 7862 and 31 and lesser methylation at the other positions. Representative electropherograms are shown in Figure S1.

### Confirmation of the presence of the viral transcript *E7-E1∧E4* that encodes repressor E2 in CaCx cases based on APOT-coupled-quantitative-RT-PCR assay and classification of samples into episomal (pure or concomitant) and purely integrated viral genomic forms

**Figure 2:**
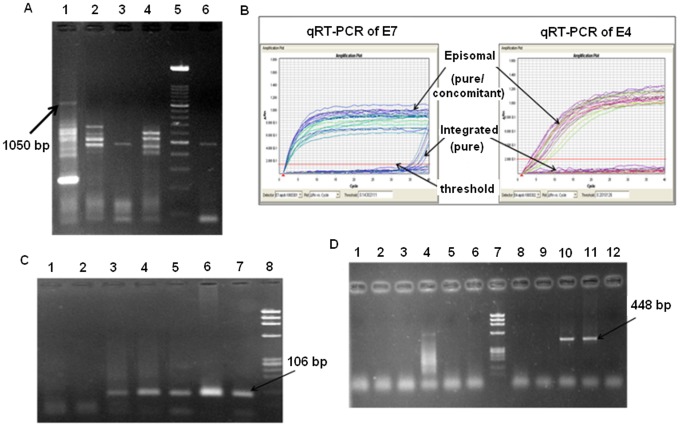
APOT-coupled-quantitative-RT-PCR assay. (**A**) Representative gel electrophoresis showing P2 - (dT)17-P3 products. Lane 1: sample showing presence of 1050 bp band-size indicating episomal viral genome. Presence of other band-sizes indicates concomitant status; Lanes 2, 3, 4, 6: absence of band-size 1050 bp indicates integrated status of these samples. Lane 5: 100 bp ladder (Roche)**.** (**B**) TaqMan-based quantitative RT-PCR amplification plots of *E7* and *E4* cDNA. *E7* is transcribed in both episomal (pure or concomitant) and integrated cases, but *E4* is transcribed in only episomal cases. (**C**) Agarose gel electrophoresis of PCR products of *GAPDH* cDNA (primers span exon-exon junction). Lane 1: negative control; Lane 2: CaSki cell line DNA (negative control); Lanes 3–7: sample cDNAs showing specific band size of 106 bp; Lanes 3 and 7 show episomal (pure or concomitant) samples T323 and T329, respectively, while, Lanes 4–6 show purely integrated samples T327, T326 and T328, respectively; Lane 8: *Hae* III digested ?x174 DNA marker (Promega). (**D**) Agarose gel electrophoresis of PCR products of *TP53* cDNA (primers span introns). Lane 1–6, 8, 9: sample cDNA not showing specific band size of 448 bp; Lanes 1, 2, 4, 6 and 9 show purely integrated samples T326, T327, T345, T344 and T328, respectively, while, lanes 3, 5 and 8 show episomal (pure or concomitant) samples T329, T323 and T339, respectively; Lane 10: CaSki cell line DNA (positive control); Lane 11: sample DNA (positive control); Lane 12: water (negative control); Lane 7: *Hae* III digested ?x174 DNA marker (Promega).

Having confirmed the occurrence of methylation in CpGs within E2 binding sites adjacent to p97 promoter in cases harboring intact *E2* and absence in cases harboring disrupted *E2*, our next objective was to confirm the presence of E7-E1∧E4 transcripts in the former to confirm the expression of repressor *E2* transcripts in such cases in contrast to those cases having disrupted *E2*. We employed the APOT assay in which case, presence of the band size of 1050 bp upon electrophoresis of PCR products obtained with P2 and (dT)17-P3 primers in 1.5% agarose gel, (Figure 2A), was considered as specific for the repressor *E2*-splice variant, *E7-E1∧E4* ***,*** coded from intact *E2* gene. Absence of the 1050 bp band and presence of bands of lengths other than 1050 bp indicate presence of integrate-derived transcripts [19]. However, presence of 1050 bp band together with the off-sized bands indicate the presence of mixed or concomitant forms i.e., co-existence of episomal and integrated forms of the viral genomes. Such analysis identified 22 CaCx cases portraying the presence of *E7-E1∧*E4 transcript and hence the E2 repressor transcripts and 19 CaCx cases lacking the presence of this transcript. The CaCx cases could thus be speculated as harboring episomal (n = 4), concomitant (n = 18) and integrated (n = 19) forms of viral genomes, which we confirmed subsequently by performing real-time PCRs (Taqman assay) using the PCR products obtained with P2 and (dT)17-P3 primers, corresponding to *E7* and *E4* expression, instead of Southern hybridization.

The real-time PCR (RT-PCR) data corresponding to *E7* and *E4* (Figure 2B) have been represented as C_T_ values, where C_T_ is defined as the threshold cycle of PCR at which, the amplified product was first detected. *E7*- and *E4*-specific PCR efficiencies did not differ based on absolute quantification by standard curve method using different copy numbers (1.75×10^8^, 1.75×10^6^ and 1.75×10^4^ copies) of pUC19 plasmid vector with HPV16 reference sequence insert (Figure S2). Presence of mRNA was confirmed by PCR with primers spanning exon-exon junction of *GAPDH* (Figure 2C) and absence of DNA contamination was confirmed by PCR with primers spanning introns of *TP53* (Figure 2D).

According to APOT-coupled-quantitative-RT-PCR assay, the genomic status of HPV 16 could also be interpreted on the basis of the ratio, *E7* C_T_/*E4* C_T_. The CaCx samples (19/41) showing *E7* and *ACTB* expression but no *E4* expression (*E7* C_T_/*E4* C_T_ = undetermined), were designated as cases with integrated viral genomes. CaCx samples (18/41) showing more expression of *E7* than *E4* (*E7* C_T_/*E4* C_T_≤1) along with *ACTB* expression, were regarded as cases portraying the presence of both episomal and integrated viral genomes (concomitant). There were four CaCx cases showing similar levels of expression of both *E7* and *E4* (*E7* C_T_/*E4* C_T_ = 1) along with *ACTB* expression, and these were regarded as samples harboring episomal viral genomes. There was no significant (p = 0.186; t-test) difference in *ACTB* mRNA expression between CaCx cases harboring episomal viral genomes (pure and concomitant) with mean±sd = 32.98±3.99 and purely integrated viral genomes with mean±sd = 34.99±3.26. Thus, 53.66% (22/41) of CaCx cases harboured intact viral genomes of which, 18.18% (4/22) had pure episomal genomes and 81.82% (18/22) had concomitant genomes (Table 1).

**Table 1:** Classification of samples with intact *E2* gene into those harbouring pure episomal and concomitant (episomal+integrated) viral genomes based on *E7* C_T_/*E4* C_T_ derived from APOT-coupled-quantitative-RT-PCR assay.

Serial number	Sample ID	*E7* C_T_/*E4* C_T_(APOT-coupled-quantitative-RT-PCR)	Viral genome status
**1**	T315	0.118	concomitant
**2**	T318	0.138	concomitant
**3**	T323	1.011	pure episomal
**4**	T324	0.554	concomitant
**5**	T325	1.087	pure episomal
**6**	T329	0.089	concomitant
**7**	T331	0.086	concomitant
**8**	T339	1.029	pure episomal
**9**	T215	0.297	concomitant
**10**	T222	0.515	concomitant
**11**	T210	0.285	concomitant
**12**	T217	0.422	concomitant
**13**	T234	0.419	concomitant
**14**	T170	0.428	concomitant
**15**	T184	0.227	concomitant
**16**	T226	0.453	concomitant
**17**	T171	0.446	concomitant
**18**	T168	0.373	concomitant
**19**	T340	0.088	concomitant
**20**	T342	0.215	concomitant
**21**	T181	0.440	concomitant
**22**	T164	1.118	pure episomal

Concomitant refers to the presence of both episomal and integrated viral genomes.

Reanalysis of our data on E2BSI methylation at nt 58 in LCR on this subset of samples, also revealed that such methylation was significantly (p_trend_ = 0.001) higher among the cases portraying episomal (pure and concomitant) viral genomes (17/22; 77.27%) compared to those with purely integrated viral genomes (5/19; 26.32%).

### Analysis of the expression of *E7* and *E2* genes in CaCx cases harboring episomal (pure and concomitant) or purely integrated HPV16 genomes and correlation with viral load

Our next objective was to investigate whether the two types of cancers, (i) those harboring episomal (pure and concomitant) and (ii) integrated viral forms, differed in viral oncogenic expression, by analyzing *E7* mRNAs. We quantified *E7* expression (normalized by *ACTB*) by TaqMan-based qRT-PCR of cDNA products generated directly from the mRNAs, in a subset of 17 episomal (purely episomal and concomitant) and 13 purely integrated cases.

*E7* mRNA expression (*E7* C_T_/*ACTB* C_T_) was found to be normally distributed in both episomal (Kolmogorov-Smirnov Z value = 0.418, p = 0.995) and integrated (Kolmogorov-Smirnov Z value = 1.06, p = 0.211) cases. The ratio, *E7* C_T_/*ACTB* C_T_, was significantly lower (p<0.001; t-test) among episomal (mean±sd = 0.84±0.15) than integrated (mean±sd = 1.12±0.18) cases indicating higher *E7* expression in the former.

Relative quantification based on comparative C_T_ method also revealed the significant difference (p<0.001; Mann-Whitney U test) between ΔC_T_ (*E7* C_T_ – *ACTB* C_T_) values of cases with integrated viral genomes (median ΔC_T_ = 1.26) and with episomal genomes (median ΔC_T_ = −4.97). The fold-change analysis (using 2^−ΔΔCT^, where ΔΔC_T_ = median ΔC_T_ of episomal - median ΔC_T_ of integrated), depicted that *E7* expression in cases with episomal (pure and concomitant) viral genomes was 75.087 folds higher than cases with purely integrated viral genomes.

**Figure 3:**
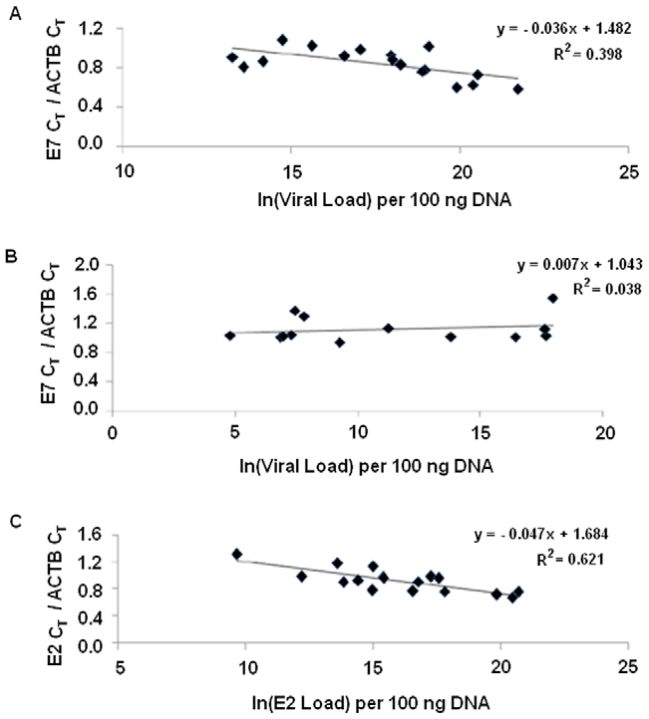
Linear regression analyses. (**A**) Correlation of *E7* C_T_/*ACTB* C_T_ with viral load (natural log values) in CaCx cases with episomal (purely episomal and concomitant) viral genomes; (**B**) Correlation of *E7* C_T_/*ACTB* C_T_ with viral load (natural log values) in CaCx cases with integrated viral genomes; (**C**) Correlation of *E2* C_T_/*ACTB* C_T_ with viral load (natural log values) with respect to *E2* gene in CaCx cases with episomal (purely episomal and concomitant) viral genomes.

Viral copy numbers (natural log transformed) per 100 ng genomic DNA were also significantly higher (p<0.001; Mann-Whitney U test) among the 17 episomal cases (median ln(viral load) = 18.02 per 100 ng DNA) compared to the 13 purely integrated cases (median ln(viral load) = 9.28 per 100 ng DNA). The ratio, *E7* C_T_/*ACTB* C_T_, was found to be significantly correlated with the viral load (p = 0.007; R^2^ = 0.398) within the episomal samples (pure and concomitant) (Figure 3A), but not within the purely integrated samples (p = 0.51; R^2^ = 0.038) (Figure 3B).

The episomal (pure and concomitant) cases showed simultaneous expression of *E2* mRNA (mean (*E2* C_T_/*ACTB* C_T_)±sd = 0.92±0.18), while the purely integrated cases failed to do so. The ratio, *E2* C_T_/*ACTB* C_T_, was found to be significantly correlated (p<0.001; R^2^ = 0.621) (Figure 3C) with *E2* gene copy numbers (median ln (*E2* load) = 15.41 per 100 ng DNA) among such cases harboring episomal (pure and concomitant) viral genomes.

### Joint analysis of *E7*C_T_ values from APOT-coupled-quantitative-RT-PCR assay and viral load among CaCx samples portraying purely integrated HPV16 genomes employing *k*-means clustering

**Figure 4:**
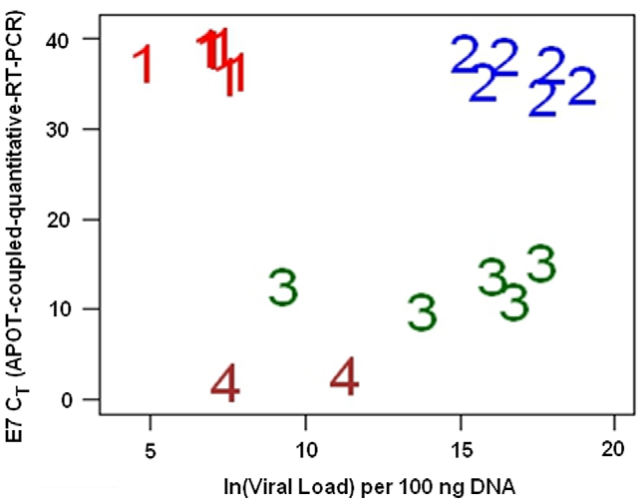
Samples with purely integrated viral genomes grouped into 4 clusters after *k*-means clustering analysis. Cluster 1: low viral load and low *E7* expression; Cluster 2: high viral load and low *E7* expression; Cluster 3: moderate viral load and moderate *E7* expression; Cluster 4: low to moderate viral load and high *E7* expression.

The lack of correlation of *E7* expression with viral load, among CaCx cases with integrated viral genomes, pointed towards the possibility of existence of heterogeneity among such CaCx cases with respect to *E7* expression. We thus further analyzed our data by performing a cluster analysis (*k*-means clustering) based on viral load and *E7* C_T_ (APOT-coupled-quantitative-RT-PCR). Such analysis could optimally classify the cases with disrupted viral genomes (46.34%; 19/41) into four clusters (Figure 4) as depicted in Table 2. The respective cluster-centres have been represented in Table 3. The *ACTB* mRNA expression of these cases did not correlate with either of their viral load (p = 0.588; linear regression), or *E7* C_T_ values (APOT-coupled-quantitative-RT-PCR) (p = 0.753; linear regression).

**Table 2:** *k*-means clustering of CaCx cases portraying purely integrated HPV16 genomes.

Serial number	Sample ID	ln(Viral Load) per 100 ng DNA	*E7* C_T_(APOT-coupled-quantitative-RT-PCR)	Cluster	Distance from cluster centre
**1**	T330	4.8	37.53	1	2.108
**2**	T267	7.83	36.56	1	1.656
**3**	T259	6.86	39.11	1	1.198
**4**	T263	7.3	39.34	1	1.491
**5**	T261	7.48	36.02	1	1.987
**6**	T228	6.97	38.91	1	1.003
**7**	T326	17.71	33.65	2	2.817
**8**	T265	16.46	38.28	2	1.979
**9**	T156	18.97	35.07	2	2.355
**10**	T239	15.77	35.48	2	1.535
**11**	T233	15.18	38.52	2	2.818
**12**	T244	17.99	37.28	2	1.328
**13**	T328	17.66	15.18	3	3.996
**14**	T336	13.81	9.8	3	2.825
**15**	T345	9.28	12.7	3	5.445
**16**	T333	16.78	11.01	3	2.527
**17**	T344	16.07	13.68	3	1.81
**18**	T321	11.26	2.74	4	1.933
**19**	T327	7.45	2.08	4	1.933

***Cluster 1*** *: samples with low viral load, low E7 expression; **Cluster 2:** samples with high viral load, low E7 expression; **Cluster 3:** samples with moderate viral load, moderate E7 expression; **Cluster 4:** samples with low to moderate viral load, high E7 expression.*

**Table 3:** Final cluster centers obtained from *k*-means clustering of purely integrated samples.

Variables	Clusters			
	1	2	3	4
Viral Load†	6.87	17.01	14.72	9.36
*E7* C_T_ *	37.91	36.38	12.47	2.41

* *E7 C_T_ was derived from APOT-coupled-quantitative-RT-PCR assay.*

† *viral load values were transformed to natural log values.*

Of these four clusters, Cluster 1 (mean viral load±sd = 6.87±1.074; mean *E7* C_T_±sd = 37.91±1.416) included 6 samples (31.60% of purely integrated samples). Overall, these had low viral load and low *E7* expression. Cluster 2 (mean viral load±sd = 17.01±1.447; mean *E7* C_T_±sd = 36.38±1.948) also included 6 samples (31.60% of purely integrated samples), which had high viral load and low *E7* expression. Among these samples, *ACTB* transcription (with the same amount of cDNA as that used in APOT-coupled-quantitative-RT-PCR analysis for *E7* and *E4*) did not correlate with viral load (p = 0.942; linear regression). Cluster 3 (mean viral load±sd = 14.72±3.359; mean *E7* C_T_±sd = 12.47±2.128) included 5 samples (26.32% of purely integrated samples) and these portrayed moderate viral load and moderate *E7* expression. Cluster 4 (mean viral load±sd = 9.36±2.694; mean *E7* C_T_±sd = 2.41±0.467) included 2 samples (10.56% of purely integrated samples), which revealed low to moderate viral load and very high *E7* expression. The distances of samples from the centers of their corresponding clusters are provided in Table 2. Thus of the four clusters, two were found to be characterized by moderate to high *E7* expression and the remaining two depicted low *E7* expression on the basis of APOT-coupled-quantitative-RT-PCR assays. Mean C_T_-values for *ACTB* expression did not differ among the four clusters (p = 0.19; ANOVA).

## Discussion

Opposed to the prevailing concept of HPV16 *E2* gene disruption as a consequence of viral integration into the host genome, recent reports, including our study identified the presence of intact *E2* genes in a large number of CaCx cases prompting us to explore new paradigms of cervical carcinogenesis [7], [9], [10], [12], [22], [23]. Herein therefore, we explored alternative mechanisms of loss of E2 repression that could lead to sustained *E6*/*E7* expression even in presence of episomal viral genomes (pure or concomitant) with intact *E2* gene.

Earlier, we identified that E2BS-I methylation at nucleotide 58 within LCR, was significantly higher among HPV16 positive CaCx cases harboring intact *E2*, compared to HPV16 positive controls [9]. Our present study restricted to CaCx cases only and an enhanced sample size, also revealed initially, overrepresentation of methylation at nucleotide 58 within E2BS-I in LCR among *E2*-intact, compared to *E2*-disrupted cases. We confirmed this finding subsequently in HPV16 positive CaCx cases harboring episomal (pure and concomitant) viral genomes with intact *E2* gene as compared to those with purely integrated viral genomes with disrupted *E2* gene. Sequence analysis of a subset of the samples further revealed that methylation was also prominent among *E2*-intact cases at CpGs in positions 31, which is SpI binding site, 37 and 43, which are within E2BS-II, and 52, which is within the E2BS-I. This observation further substantiates the role of E2BS-I and II methylation in blocking E2 repressor activity in cases harboring intact *E2*. This is supported by the *in vitro* analysis that HPV16 E2 binding site methylation prevents E2 binding [24], [25]. Studies by Fernandez *et al.*, 2009, and Brandsma *et al.*, 2009, also made similar observations [26], [27].

In this study, we also confirmed the presence of the major viral transcript (*E7-E1∧E4*) derived from episomal viral DNA (1050 bp in size, which results in the complete repressor *E2* transcript), in a subset of the CaCx cases selected randomly, by an improved APOT assay. This assay involved coupling of the APOT assay with quantitative RT-PCR for *E7* and *E4* (nested to *E2* gene) transcript confirmation, instead of employing Southern hybridization. On the basis of the ratio, *E7* C_T_/*E4* C_T_, derived from APOT-coupled-quantitative-RT-PCR, the viral genomic forms could be categorized into pure episomal, concomitant (episomal and integrated) and pure integrated. This assay revealed *E7* expression in all cases irrespective of *E2*-status, but *E4* expression in only a subset of cases, which we considered to harbor episomal (pure episome or concomitant) viral genomes. It also indicated that *E7* expression was quantitatively several folds higher among CaCx cases with episomal HPV16 genomes over those with integrated viral genomes (Figure 2B). The difference in normalized *E7* mRNA expression turned out to be 75.087 folds higher in episomal (pure or concomitant) cases compared to the purely integrated cases.

Our finding is further consolidated by the observations from another study suggesting no significant increase in *E6* or *E7* expression following *E2*-disruption [28]. Our observation is strengthened by the fact that we recorded a significant correlation of *E7* expression with viral load and *E2* expression with *E2* gene copy numbers in cases with episomal (pure or concomitant) HPV16 genomes, as opposed to those with purely integrated viral genomes. This ensured the expression of *E2* from all viral genomes that were episomal and harbored intact *E2* genes. A number of other studies have also observed the expression of *E2* from *E2*-intact viral genomes [26], [27], [29], [30]. Alloul and Sherman, 1999, have also recorded the translation of E2 from such transcripts by *in vitro* and *in vivo* experiments, suggesting that E2 could also be translated in tumor samples [31]. Fernandez *et al*., 2009, based on chromatin immunoprecipitation (ChIP), also showed that the E2 viral protein could not bind to the methylated E2 binding sites within the LCR region, thereby resulting in *E6* and *E7* overexpression and this could be reversed by a DNA demethylating agent (5-aza-29-deoxycytidine) [26]. Such findings, together with our observation of *E2* expression concomitant with the expression of *E7*, further strengthens the biological plausibility of E2BS-I and II methylation, specifically at nucleotide 58 adjacent to the p97 promoter, as a key alternative mechanism of loss of E2 repressor activity in CaCx cases harboring episomal viral genomes with intact *E2* genes.

Apart from functioning as a transcriptional regulatory factor for viral oncogene expression, E2 also acts in assisting the assembly of E1 on the viral origin, thereby facilitating viral genome replication and segregation by tethering the viral genomes to host mitotic chromosomes [17]. In this study, we also justified the biological plausibility of E2 in viral replication and segregation, by recording E2 expression among CaCx cases harboring episomal viral genomes, which was concomitant with enhanced viral load and lack of methylation at the viral replication origin, i.e. nucleotide 7862, compared to cases with integrated viral genomes. Taken together, we propose that one other pathway of attaining enhanced expression of *E6* and *E7* in CaCx cases harboring episomal HPV16 genomes with intact *E2* could likely be the ability to mount high viral copy numbers. It may further be speculated that a high copy number in the episomal form also essentially ensures that each dividing cell gets its share of virus, in contrast to the integrated form within host genome, where each cell gets its share of the virus along with the host chromosomes, despite low viral load.

Viral integration, by non-homologous end-joining recombination, mainly involves the hinge region of *E2* ORF with deletion of few nucleotides downstream [32]. This disrupts negative feedback control by E2 repressor on oncogenic expression [33]. *E6*/*E7* transcripts from integrated DNA capture 3′ cellular polyadenylation signals thereby increasing viral mRNA stability [34], [35]. In HPV16 infected CaSki cell line, about 500–600 copies of viral genome are integrated at about 12 chromosomal sites, mostly in tandem arrays, also called ‘concatemers’ [36]. The 3′ repeat of the tandem array remains transcriptionally active, while others are silenced by methylation [37], [38]. Such facts, impressed upon us to interpret our failure to record a correlation between *E7* expression and viral copy numbers among the CaCx cases having integrated viral genomes, as revealed by application of *k*-means cluster analysis considering *E7* C_T_ from the APOT-coupled-quantitative-RT-PCR assay, in conjunction with viral load. As depicted in Figure 4, the cluster 1 samples (low viral load and low *E7* expression) could be speculated as harboring single-copy integrated forms of HPV16. The cluster 2 samples (high viral load and low *E7* expression) could be interpreted as concatenated forms of HPV16. Cluster 3 samples (moderate viral load and moderate *E7* expression), on the other-hand, could be speculated as representing many single-copy integrated viral forms cumulatively expressing *E7* mRNA. Likewise, the cluster 4 samples (low to moderate viral load and high *E7* expression) could be speculated as harboring viral genomes that were single copy integrates at few sites and likely to be under the effect of strong host promoters accounting for the enhanced *E7* mRNA expression. There was no difference in the *ACTB* mRNA expression among the four clusters of samples.

Thus, based on APOT-coupled-quantitative-RT-PCR assay along with viral load, it has been possible not only to distinguish between episomal and integrated CaCx cases, but also to identify variations in the oncogene expression levels of these two groups of samples. Moreover, this is the first report of its kind to reveal that amongst the CaCx cases harboring purely integrated HPV16 genomes, there exists immense diversity in terms of viral physical status and viral load as well. Worth noting is the fact that 60% of the CaCx samples harboring purely integrated viral genomes appeared to represent the single-copy integrations at few sites (similar to SiHa cell line) and concatenated forms (similar to CaSki cell line), while the remaining appeared to deviate from such canonical forms that have mostly been reported in earlier studies. This assay further revealed that majority of CaCx cases with episomal viral genomes harboured concomitant forms (81.81%) (Table 1).

Integration of viral genomes into host genome is mostly associated with the disruption of *E2* ORF in the region that codes for ‘hinge region’ of the E2 protein [20]. Therefore, due to integration, 3′ end of the hinge is most frequently lost along with the loss of the early polyadenylation signal of the viral genome [38]. In the present study, *E4*-specific primer-probe set was designed in a way such that the amplicon (nucleotides 3439–3556) encompasses the 3′end of the hinge (nucleotides 3418-3542). This ensured amplification of *E4*, only in presence of intact hinge (that is intact repressor-coding *E2*). In the traditional APOT assay, the *E4* probe (nucleotides 3449–3472) used for Southern Blot does not encompass the 3′ end of the hinge and cannot identify integrate derived fusion transcripts resulting from partial *E4* disruption [19]. However, our TaqMan-based qRT-PCR could confirm *E7* and *E4* transcripts, either to reveal the presence of *E7-E1∧E4* splice variant (that produces repressor E2) or disruption of *E1∧E4* (that fails to produce repressor E2) and hence appears to be highly specific over the traditional APOT assay.

In the APOT-coupled-quantitative-RT-PCR assay, to overcome intra-sample variation between *E7* and *E4* real time PCRs, we designed duplex PCRs with differential fluorochrome tags for *E7* (FAM-tagged) and *E4* (VIC-tagged) probes. To account for the inter-sample variation, C_T_ values from RT-PCR (TaqMan) of *ACTB* gene were compared between episomal (pure or concomitant) and purely integrated cases and were found to be similar. *ACTB* gene expression also did not correlate with either viral load or *E7* expression (APOT-coupled-quantitative-RT-PCR) among the cases harbouring purely integrated viral genomes. *ACTB* mRNA expression also did not differ significantly among the four clusters of purely integrated cases obtained from *k*-means clustering. Thus, nested PCR based APOT-coupled-quantitative-RT-PCR assay increased the sensitivity of capturing the entire spectrum of *E7* mRNA expression level (ranging from C_T_ = 2.08 to C_T_ = 39.34 among the 19 purely integrated samples), which otherwise could not be detected by the *E7* expression assay using the cDNAs directly from the corresponding mRNAs (Figure 3B).

Our study further revealed that detection of integration status of viral genomes on the basis of DNA analysis only [7], [12] could misclassify concatenated forms as *E2*-intact genomes (cluster 2 of Table 2). However, this could be overcome by employing the transcript based APOT-coupled-quantitative-RT-PCR assay developed by us. Moreover, by estimating oncogenic mRNA expression by *E7*-specific probe instead of *E6*-specific probe, we could also avoid the possible complication in probe hybridization due to occurrence of various *E6* mRNA transcripts (E6*I, E6*II, *E6–E7*) [39]. Thus, this novel “APOT-coupled-quantitative-RT-PCR assay” could serve as an essential tool in mapping the heterogeneity of the HPV16 genomes in CaCx cases in conjunction with viral load.

## Conclusion

This study has provided novel insights into HPV16 related CaCx pathogenesis. High *E7* expression in CaCx cases having episomal (pure episomes or concomitant) viral genomes with intact *E2* could be attributable to loss of E2 repressor activity due to E2BS-I/II methylations. High viral load among CaCx cases having episomal (pure and concomitant) viral genomes as compared to those with purely integrated viral genomes could be attributable to the potential of E2 in maintenance of replication and segregation of viral genomes in such cases. The underlying mechanism of cervical carcinogenesis under the impact of oncogenic HPV is mediated through protein-protein interactions of the host and virus. We therefore speculate that HPV16 positive CaCx cases that harbour episomal viral genomes with intact *E2* are likely to be distinct biologically, from the purely integrated viral genomes in terms of host genes and/or pathways involved in cervical carcinogenesis. Moreover, the novel technique, APOT-coupled-quantitative-RT-PCR assay, together with viral load could be used to classify CaCx samples into various groups based on different viral genomic forms. Such findings therefore prompt towards undertaking studies to decipher the role of host genomic underpinnings, if any, in influencing the status of the viral genomes within the host cells and their impact on disease prognosis.

## Supporting Information

Figure S1 **Representative electropherograms (bisulphite sequencing) showing methylation status of CpGs within viral LCR regions. Nucleotide (nt) position 31 is SpI binding site, nt 37 and 43 are E2BS-I and nt 52 and 58 are E2BS-II.** The upper two panels show sequences of two *E2*-intact patient specimens (T241 & T256) and the lower two panels show sequences from *E2*-disrupted samples (T122 & SST30), where all Cs have converted to Ts.
(TIF)

Figure S2 **Standard Curve Plots of** ***E7*** **and** ***E4*** **RT-PCR based on Absolute Quantification of HPV16 plasmid DNA.** (A) *E7* based qRT-PCR (B) *E4* based qRT-PCR. Both *E7* and *E4* have similar slopes, which justify their similar efficiencies. The three standards used were respectively, 1.75×10^8^, 1.75×10^6^ and 1.75×10^4^ copies of HPV16 plasmid (pUC19 plasmid vector with HPV16 reference sequence insert)
(TIF)
