# Supplementary figures and images for: Some Novel Insights on HPV16 Related Cervical Cancer Pathogenesis Based on Analyses of LCR Methylation, Viral Load, E7 and E2/E4 Expressions
Source: PLoS One. 2012 Sep 6;7(9):e44678. doi: 10.1371/journal.pone.0044678 (PMC3435323; doi:10.1371/journal.pone.0044678)

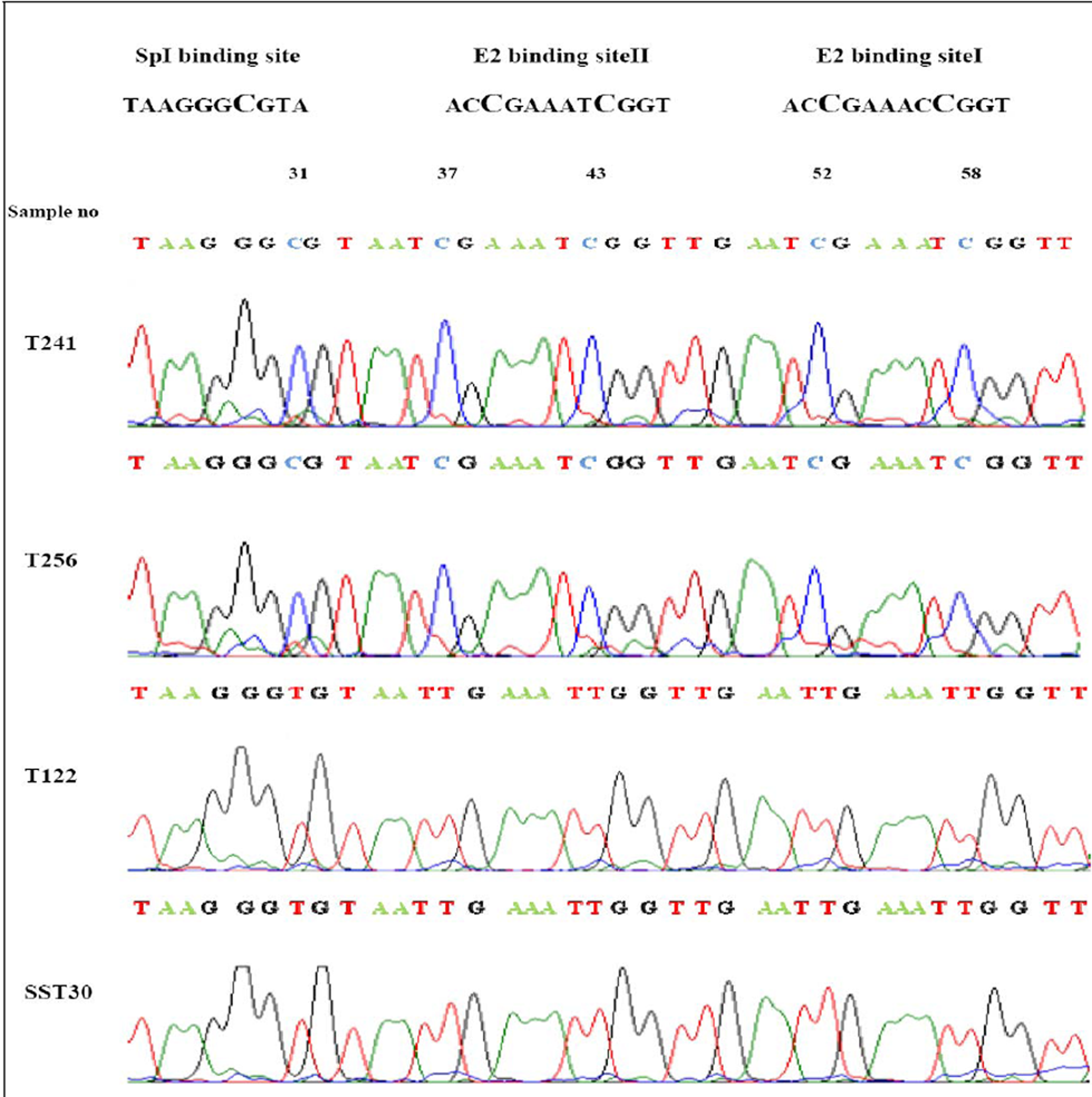

Supplement: Figure S1 — Representative electropherograms (bisulphite sequencing) showing methylation status of CpGs within viral LCR regions. Nucleotide (nt) position 31 is SpI binding site, nt 37 and 43 are E2BS-I and nt 52 and 58 are E2BS-II. The upper two panels show sequences of two E2-intact patient specimens (T241 & T256) and the lower two panels show sequences from E2-disrupted samples (T122 & SST30), where all Cs have converted to Ts. (TIF) [file pone.0044678.s001.tif]

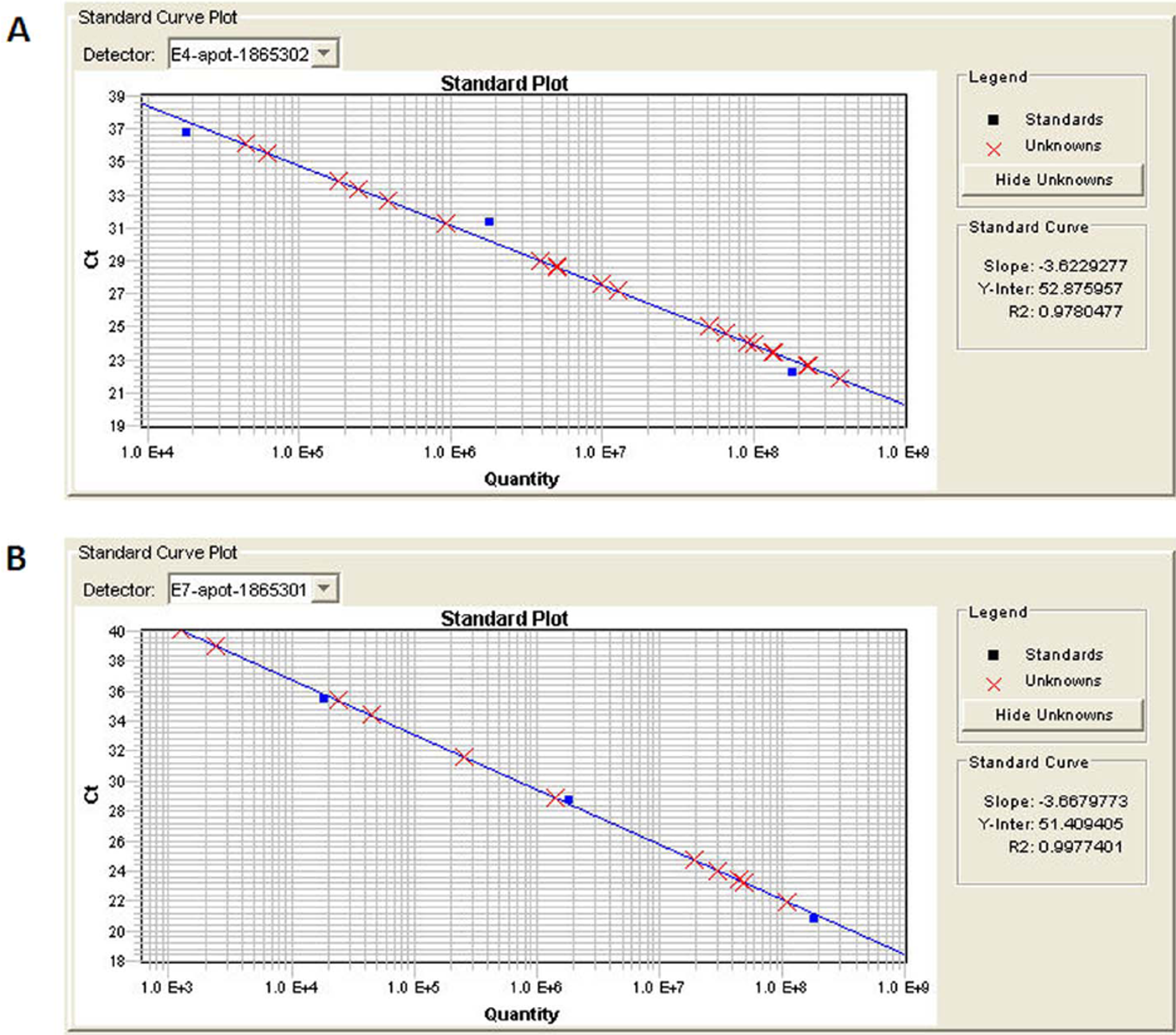

Supplement: Figure S2 — Standard Curve Plots of E7 and E4 RT-PCR based on Absolute Quantification of HPV16 plasmid DNA. (A) E7 based qRT-PCR (B) E4 based qRT-PCR. Both E7 and E4 have similar slopes, which justify their similar efficiencies. The three standards used were respectively, 1.75×10^8^, 1.75×10^6^ and 1.75×10^4^ copies of HPV16 plasmid (pUC19 plasmid vector with HPV16 reference sequence insert) (TIF) [file pone.0044678.s002.tif]
